# Barriers and facilitators to implementing a cancer risk assessment tool (QCancer) in primary care: a qualitative study

**DOI:** 10.1017/S1463423621000281

**Published:** 2021-10-07

**Authors:** Joseph N. A. Akanuwe, Sharon Black, Sara Owen, Aloysius Niroshan Siriwardena

**Affiliations:** 1 Community and Health Research Unit, School of Health and Social Care, University of Lincoln, Lincoln, UK; 2 Nottingham Trent University, Nottingham, UK

**Keywords:** cancer risk assessment tools, implementing, primary care, QCancer

## Abstract

**Aim:**

We aimed to explore service users’ and primary care practitioners’ perspectives on the barriers and facilitators to implementing a cancer risk assessment tool (RAT), QCancer, in general practice consultations.

**Background:**

Cancer RATs, including QCancer, are designed to estimate the chances of previously undiagnosed cancer in symptomatic individuals. Little is known about the barriers and facilitators to implementing cancer RATs in primary care consultations.

**Methods:**

We used a qualitative design, conducting semi-structured individual interviews and focus groups with a convenience sample of service users and primary care practitioners.

**Findings:**

In all, 36 participants (19 service users, 17 practitioners) living in Lincolnshire, were included in the interviews and focus groups. Before asking for their views, participants were introduced to QCancer and shown an example of how it estimated cancer risk. Participants identified barriers to implementing the tool namely: additional consultation time; unnecessary worry; potential for over-referral; practitioner scepticism; need for training on use of the tool; need for evidence of effectiveness; and need to integrate the tool in general practice systems. Participants also identified facilitators to implementing the tool as: supporting decision-making; modifying health behaviours; improving speed of referral; and personalising care.

**Conclusions:**

The barriers and facilitators identified should be considered when seeking to implement QCancer in primary care. In addition, further evidence is needed that the use of this tool improves diagnosis rates without an unacceptable increase in harm from unnecessary investigation.

## Introduction

Primary care, particularly in the UK, plays an important role in diagnosis of cancer, as general practitioners (GPs) conduct initial clinical assessment and refer to specialist care for suspected cancer. Despite this important role played by GPs, the UK has one of the lowest cancer survival rates among high-income countries (Cancer Research UK, 2019; Arnold *et al.*, 2019). This may be due to patients presenting late with symptoms, GPs failing to recognise potential cancer symptoms during primary care consultations or delayed referral to specialist care (Al-Azri, 2016; Bowen and Rayner, 2002; Koyi *et al.*, 2002).

Cancer risk assessment tools (RATs) designed for symptomatic individuals have been recommended for implementation in primary care to estimate an individual’s risk of developing cancer based on their risk factors and symptoms, to enable earlier detection or diagnosis of the condition (Hamilton, 2009; Hippisley-Cox and Coupland, 2013). Two cancer RATs designed for symptomatic patients presenting to primary care are currently being promoted in UK general practice: QCancer (Hippisley-Cox and Coupland, 2013) and the RAT (Hamilton, 2009).

QCancer has been independently validated (Collins and Althman, 2012; Collins and Althman, 2013) and found to accurately predict risk of cancer in primary care. However, little is known about service users’ and practitioners’ views on this tool in terms of the perceived barriers and facilitators to its implementation. Our aim was to explore what service users (adults without a cancer) and primary care practitioners (GPs and practice nurses) perceived as barriers and facilitators to the implementation of QCancer in primary care consultations.

## Methods

We used the same methodological approach for this study as in a recently published study, (Akanuwe *et al.*, 2020) because both studies arose from one research project based on the same design.

We used semi-structured face-to-face individual interviews and focus groups to collect qualitative data from participants in Lincolnshire in the East Midlands region of England. The School of Health and Social Care Ethics Committee at the University of Lincoln granted ethics approval for the study.

We recruited a convenience sample of service users without cancer and primary care practitioners who agreed to participate in the study. Practitioners were offered standard backfill costs for participating in the interview. Patients with cancer were not recruited because RATs are not indicated for this group, and we did not recruit those who had been referred, were undergoing assessment or might have been concerned about a risk of cancer because they had recently consulted with symptoms, and because discussion about diagnosis may have been stressful for this group even if they could provide information.

The interview schedule seeking barriers and facilitators to implementation of the tool was informed by a theoretical framework, the Consolidated Framework for Implementation Research (CFIR), (Damschroder *et al.*, 2009) which seeks to understand human and other factors involved in the deployment of innovations such as cancer RATs. The data analysis and interpretation were also informed by relevant constructs within the CFIR (Damschroder *et al.*, 2009) including: relative advantage; patients’ needs and resources; compatibility; knowledge and beliefs of individuals involved; and reflecting and evaluating, which could be facilitators or barriers to implementation.

*Relative advantage* refers to practitioners’ greater willingness to use cancer RATs if they are perceived to have advantages over existing alternatives such as guidelines. The extent to which *patients’ needs and resources* are enhanced by use of cancer RATs is another important factor in implementation. ‘Compatibility’ describes how cancer RATs fit with existing workflows and systems and how they align with individuals’ own norms, values, perceived risks and needs. Knowledge and beliefs include how much potential users know about, are skilled in using and feel about a cancer RAT, which will inevitably affect their enthusiasm for it. *Reflecting and evaluating* refer to feedback from and outcomes of using the tool in practice.

Participants were recruited using flyers in local public places (e.g. the public library, advertisements on notice boards) and through members of a patient and public involvement group. Service users were offered individual interviews because these were considered more appropriate for discussion of sensitive information related to risk factors. Service users who were willing to be interviewed contacted the researcher (JA) for more information and an appointment for a face-to-face interview in a location of their choice, either their own home or at the university, was provided.

Following invitation letters sent to practitioners at their general practices, those interested to take part contacted the researcher for more information and an appointment with the researcher (JA) for either an individual interview or focus group depending on preference. Individual interviews provided in-depth discussion with participants, while focus groups enabled interactions in practice teams, both providing rich data.

Participants gave a written consent and were assured they could discontinue at any point. All participants gave permission for audio-recording, and notes were taken to complement audio-recorded data. Before asking for participants’ views, a vignette of the QCancer tool was shown, explained and demonstrated, either on computer or as a paper version.

After transcribing data verbatim, the framework approach (Ritchie and Spencer, 1994) was used for analysis facilitated by NVivo version 10. A priori codes, which informed the interview guide, formed an initial coding framework, and further inductive codes were identified as analysis of the interview data progressed. Two investigators (JNA and ANS) read the transcripts thoroughly and derived the initial coding framework which was discussed and agreed by the research team. Through further interpretation and discussion, initial themes were developed iteratively into a smaller number of overarching themes.

Data collection ceased when analysis showed saturation, meaning no new codes or themes were generated (Hennink *et al.*, 2016). Service user and practitioner data were analysed separately and then compared for similarities and differences. To add trustworthiness to the analysis and reporting of our research, we followed the Consolidated Criteria for Reporting Qualitative Studies (Tong *et al.*, 2007) (see Supplementary Table S1).

## Results

Nineteen service users (aged from 21 to 71 years) and 17 practitioners (aged from 33 to 55 years) were interviewed between September 2014 and September 2015. Two service users had a previous diagnosis of cancer, and all other service user participants had relatives or friends who had a previous diagnosis of cancer, which may have motivated them to participate in the study. Once they had agreed to take part, no participants dropped out of the study. Table 1 presents further details of the participant characteristics.

**Table 1.** Participant characteristics

	Service users	Practitioners
Gender		
Male	7	13
Female	12	4
Age group (years)		
20–29	3	–
30–39	4	3
40–49	1	10
50–59	3	4
60–69	5	–
70–79	3	–
Ethnicity		
White British	19	6
Indian	–	6
Pakistani	–	3
Asian British	–	1
Bangladeshi	–	1
Practice patient list size		
200–2900	–	1
3000–3900	–	–
4000–4900	–	–
5000–5900	–	–
6000–6900	–	8
7000–7900	–	–
8000–8900	–	–
9000–9900	–	8

The overarching themes identified barriers and facilitators to implementing the tool. Barriers identified were the need for additional consultation time; unnecessary worry relating to cancer investigations; over-referral that could over-burden services; practitioner scepticism; the need to train practitioners on use of the tool and the need to establish effectiveness of the tool against existing practice. These are explained below with further details of the theoretical framework, key themes, codes and quotations in Supplementary Table S2.

### Additional consultation time

In line with the CFIR constructs of readiness for implementation and patient needs and resources (Damschroder *et al.*, 2009), service users were concerned that GPs and nurses were already busy and that additional consultation time would be needed for using a cancer RAT: ‘*Practitioners in general practice would need more time to use the tool in consultations*’ (Service User 7: individual interview). Practitioners expressed similar concerns: ‘*It’s more a question of more time really, because at the moment we’re in crisis. So, we don’t want more work*’ (Practitioner 11 [GP]: focus group [FG] 2).

### Unnecessary worry relating to cancer investigations

It was felt that unnecessary worry or anxiety could be generated by an increased number of cancer investigations. This relates to the concept of ‘patient needs, and resources’ expressed within the CFIR (Damschroder *et al.*, 2009). Service users and practitioners agreed that people might worry if it was not explained to them that the tool provided a risk assessment rather than a cancer diagnosis:

*‘Some people may not understand and they can be too worried especially if they don’t explain that it is just a risk but it is not guaranteed that they will get cancer’* (Service User 11: individual interview).

*‘If you tell the patient they’ve got 1% cancer, which is creating unnecessary anxiety, they will say doctor, you said I have got 1% chance of getting cancer and you are not doing anything about it’* (Practitioner 2 [GP]: individual interview); *‘…you can probably make them more worried’* (Practitioner 16 [Practice Nurse]: FG 3).

### Over-referral and over-burdening services

In relation to the CFIR construct of patient needs and resources (Damschroder *et al.*, 2009), there were concerns from some participants that additional referrals could over-burden services:

*‘It could lead to over-referral as some people may have a certain risk but will not have cancer after they have been referred and tested’ (Service User 17: individual interview); ‘It will put a strain on the NHS; you don’t want to over burden the services as well’* (Practitioner 4 [GP]: individual interview).

In contrast, other practitioners felt that their use of clinical judgement alongside the tool to refer patients could reduce the potential for over-referral:

*‘We are not just referring but we are using our clinical judgements as well, so we would only refer those patients that need to be referred – so I don’t think there will be over-referrals’* (Practitioner 1 [GP]: FG 1).

### Practitioner scepticism

In line with the CFIR construct of knowledge and beliefs of individuals involved in the implementation process (Damschroder *et al.*, 2009) participants, particularly practitioners, felt that colleagues who had doubts about new tools may be unwilling to use them, especially if they lacked knowledge about how a tool could be used. A practitioner said: ‘*…until you said this thing, you know initially I was very sceptical about this tool’* (Practitioner 3 [GP]: individual interview).

Other practitioners were more willing to use the QCancer tool:

*‘I believe it will be good to use a cancer risk assessment tool to facilitate earlier diagnosis of cancer, and as you know, earlier diagnosis will help with earlier treatment’* (Practitioner 2 [GP]: FG 1).

### Conflict with existing guidelines

Service users felt that guidance needed to be consistent, while practitioners felt it might be confusing to use a cancer RAT with existing guidelines, for example, National Institute for Health and Care Excellent (NICE) guidelines. This relates to the constructs of complexity within the CFIR (Damschroder *et al.*, 2009). In line with this, participants stated:

*‘I think it is good for everybody to have the same sort of guidelines, so everybody should use the same sort of guidelines’* (Service User 1: individual interview).

*‘I will be quite confused about using the tool. With the NICE guidelines, you couldn’t focus on another criterion for any other risk here. I mean there are implications for investigations, referrals…, it has to be very much a repeated approach’* (Practitioner 11 [GP]: FG 2).

### High-risk symptoms need referral at any risk

Participants also felt that symptoms suggesting the presence of cancer needed to be referred for further investigation, regardless of any quantified risk using the tool:

*‘It doesn’t really matter about percentages; I know 1% is less risk. But the fact is the symptom is there, the coughing out of blood, which is quite worrying’* (Service User 13: individual interview).

*‘Regardless of what the tool said I will refer them for investigation with the symptoms. So, it doesn’t matter 1% or 0%, I will always do one thing, investigation if the symptoms are suggestive of cancer’* (Practitioner 11 [GP]: FG 2).

### Need for training on how to use the tools

Another barrier identified by practitioners was their lack of understanding on how to use the tool during a consultation, including using these correctly to calculate risk, understanding what the predicted risk meant and communicating the results to patients. They felt that training on how to use the tool in patient consultations was needed:

*‘We don’t quite understand how to use that tool. I think we need to have proper education or training on using these tools’* (Practitioner 2 [GP]: FG 1).

### Establishing effectiveness of the tools

Service users felt that the use of QCancer in patient consultations should be evaluated for effectiveness before allowing all practitioners to use them:

*‘I think if you are going to roll something out…. I would start with the doctors, see how the doctors do with it after evaluation and then move on to the practice nurses’* (Service User 12: individual interview).

Practitioners also felt that evaluating the tools would help them to compare the effectiveness of the tools with current practice: ‘*We have to make sure that it is better than what we are already doing’* (Practitioner 13 [GP]: FG 3).

Facilitators to implementation of QCancer, related to the CFIR construct of relative advantage (Damschroder *et al.*, 2009) over existing tools, included supporting clinical decision-making, modifying patient health behaviours, improving processes and speed of cancer assessment and treatment, and personalising patient care.

### Supporting clinical decision-making

Service users and practitioners expressed the view that the tool could help them to make more appropriate decisions on cancer investigations and referrals. One service user felt the tool will help to, *‘make decisions appropriately’* (Service User 1: individual interview).

A practitioner also said:

*‘I think the tool will help to guide the clinician to see the broad level of differential diagnosis. It will also facilitate referral of patients by presenting a quantitative risk value to help explain risk and make a decision’* (Practitioner 2 [GP]: FG 1).

### Modifying patient health behaviours

Although designed as a RAT for symptomatic individuals, participants felt that use of the tool could also help to identify and raise awareness about modifying health behaviours:

*‘I think it might be just raising awareness, so people realise what’s happening, and what can go wrong with them and where the risks are and may be, they can reinforce them. Where someone else like the young person who has given up smoking it might be used to reinforce by saying well, you’ve got a very low risk, so if you’ve given up smoking carry on with that. Rather than saying you’ve got a very high risk later’* (Service User 5: individual interview).

*‘I also feel the tool will help in terms of using the risk generated to advise patients who need behavioural changes. If their risk was small, I would tell them to maintain healthier lifestyles by exercising, eating a healthy diet, less alcohol and to stop smoking if they were smoking. Yes, as I said, this tool can help to empower patients to take control of their risk factors and live healthier lifestyles’* (Practitioner 2 [GP]: FG 1).

### Improving processes and speed of cancer assessment and treatment

Service users and practitioners felt the tool could facilitate earlier cancer diagnosis by improving the processes and speed of assessment and treatment:

*‘I do think it will be a useful idea, yeah. I think my first worry is that I may have cancer and most of us will like to know early so they can get it sorted. But a lot of things can be picked up, can’t they, if they spot check risk’* (Service User 4: individual interview).

*‘I think when the tool is fully integrated in our IT systems and every practitioner gets familiar with using it, it will be time saving in the long term, as the consultation, the assessments, investigations and referral processes will be faster’* (Practitioner 1 [GP]: FG 1).

### Personalising patient care

Participants felt that use of the tool would help to provide patient-centred care based on the patient’s specific cancer risks enabling a personalised rather than a more generalised plan of care:

*‘I think it will make the care more patient-centred because you’re presenting them with their own risk not a general risk, it’s personal to them and it will just make the consultation more patient focused, and I think it will make patients feel more involved in the consultation and just feel more cared for’* (Service User 12: individual interview).

*‘Patients will go away with a lot more targeted information about their personalised risk of cancer rather than a vague statement’* (Practitioner 1 [GP]: individual interview).

## Discussion

We found a range of barriers and facilitators to implementing QCancer. The barriers were the need for more consultation time, unnecessary worry and anxiety generated by cancer investigations, over-referral and over-burdening of services, practitioner scepticism about the usefulness and effectiveness of the tool, lack of training for practitioners on the use of the tool, and the need to establish the effectiveness of the tool before rolling it out in clinical practice. The facilitators related to perceptions that the tool would support decision-making, speed up the process of assessment and treatment, help to identify and modify health risk behaviours, and personalise care. This study is novel in that it analysed views of both primary care practitioners and service users. The finding that cancer RATs may help to personalise patient care and also to identify and modify health risk behaviours adds to the limited knowledge in the area of using cancer RATs.

### Strengths and limitations

This study is one of the first to elicit and compare perspectives of both service users and practitioners on QCancer, a cancer RAT designed for symptomatic individuals in general practice. Individual interviews provided information from service users and practitioners, while the focus groups facilitated discussion between practitioners in their respective general practices and both provided rich data. Another strength of this study is that data saturation was realised in terms of code (no new ideas expressed) and meaning (ideas expressed were understood) (Hennink *et al.*, 2016).

Although the study was widely publicised, all the service user participants were of White British ethnicity. People from ethnic minority groups may not have participated because of lack of awareness of the study, language problems affecting their ability to understand the advertisement or a lack of interest in participating as evidence from previous studies suggests (Gill *et al.*, 2013; Lo and Garan, 2008; Redwood and Gill, 2013).

We also acknowledge as a limitation that we did not consider differences in educational or socio-economic background of participants, particularly service users, as these factors could have informed their views of the tool. However, our focus was on comparing the views of the two groups of participants (service users and practitioners) rather than between groups of service users.

### Comparison with existing literature

The need for extra time to conduct a risk calculation and communicate this effectively, listening, informing, explaining, and discussing further investigations with the patient, is known to add complexity and costs to the patient consultation (Damschroder *et al.*, 2009). Additional time is a scarce resource in the face of increasing practitioner workload, which will affect implementation of innovations such as this. Integration of cancer RATs within general practice IT systems linked to existing patient data, with training provided on using these tools, are essential to addressing issues of time and complexity of use.

Patients feeling worried or anxious about being referred for cancer investigations was perceived as another barrier to the use of the tool, but a systematic review of randomised controlled trials of cancer RATs in primary care found no increase in cancer worry (Walker *et al.*, 2015). This contradiction may be because participants, particularly service users, who had not yet experienced using the tool, were expressing what they felt could happen if the cancer risk information was not properly communicated to them (Akanuwe *et al.*, 2020; Kim *et al.*, 2018). The views expressed in this study suggest that some patients could indeed experience worry and anxiety if their care, from investigations through to diagnosis and treatment of cancer, was not properly planned and carried out to meet their needs.

Another barrier identified by participants was over-referral. Current guidance advises that Cancer Decision Support tools should prompt primary care practitioners to think about the possibility of cancer and then decide on referral based on their clinical judgement (Macmillan Cancer Support, 2015). Although some practitioners in this study agreed that RATs should be used alongside professional judgement, only referring patients who needed this, the potential for increased rates of referral of people without cancer (false positives) remains a concern, with potential costs needing to be weighed against late referral.

Practitioners in a simulation study conducted in Australia appeared not to trust some risk outputs of the QCancer tool, (Chiang *et al.*, 2015) and this accords with scepticism expressed by some clinicians in this study. Practitioners might be sceptical because they perceive the evidence that QCancer or other cancer RATs improve outcomes is limited. Intervention characteristics can be an important potential facilitator or barrier, (Damschroder *et al.*, 2009) particularly when evidence of effectiveness in practice is lacking (Emery *et al.*, 2017). Furthermore, lack of trust in the risk calculation on the part of some GPs was a barrier to successful implementation of cancer RATs in primary care, especially when it conflicted with clinical judgement (Chiang *et al.*, 2015).

Dikomitis and colleagues found that training and guidance were needed when using cancer RATs in routine practice because of difficulties experienced by practitioners in employing the tools (Dikomitis *et al.*, 2015). Practitioners in this study were concerned about difficulties in understanding, accessing and using the cancer RAT. Indeed, to meet the needs of patients, clinicians’ learning needs around cancer RATs need to be addressed through information (Jones *et al.*, 2000; Sowden *et al.*, 2001) and training.

Another barrier was the perception that patients with symptoms suggestive of cancer would need to be referred for further investigations irrespective of their quantified risk. Indeed, it has been suggested that when using Cancer Decision Support tools, practitioners who suspect a possible cancer diagnosis can refer a patient even if their quantified risk is low or does not meet the referral NICE guidelines (Macmillan Cancer Support, 2015). Macmillan Cancer Support, who have integrated QCancer and the RAT in the electronic Cancer Decision Support (eCDS) tools, have suggested that these tools can complement existing NICE guidelines by flagging an alert on the computer screen about the possibility of cancer. Following this flagging on the computer, the clinician can then decide whether to refer a patient, based on NICE guidelines (Macmillan Cancer Support, 2015).

With reference to facilitators, participants in this study felt that the use of the cancer RAT could support decision-making especially with patients whose cancer symptoms were unclear, helping to speed up the assessment, diagnosis and treatment of cancer. This supports evidence from a previous study that the RAT helped GPs with lung and colorectal cancer symptom recognition and confirmed their decision about whether to refer (Green *et al.*, 2015). In addition, Green and colleagues found that embedding clinical decision support tools in clinical practice was more likely to be achieved when they were used to support, rather than supersede, the clinical judgement of practitioners (Green *et al.*, 2015).

Another facilitator found in this study about the use of the tool helping to identify, raise awareness of and promote positive health behaviours in patients, supports evidence from a systematic review which suggests that health promotion messages within RATs may have positive effects on behaviour change (Walker *et al.*, 2015).

Cancer RATs derive risk, based on an individual patient’s risk factors and symptoms, which helps to personalise care and determine further action, including referral and further investigations. Personalised or person-centred care is about taking into consideration the desires or values, social circumstances, and lifestyles of people, while working with people as individuals to develop appropriate solutions (Gill *et al.*, 2013; Sepucha *et al.*, 2008).

### Implications for practice and further research

Barriers to the use of the cancer RAT need to be addressed. It may be necessary to allow extra consultation time when using QCancer. Ensuring that the tool is integrated in the general practice IT system will aid its use, and training practitioners on how to access and use the tool during the patient consultations will be important. Macmillan Cancer Support and Cancer Research UK have worked with the major primary care IT providers (EMIS, SystmOne and Vision+) to integrate the eCDS tools into GP systems.

Practitioners are likely to refer patients with symptoms suggestive of cancer whatever their quantified risk if these fall within NICE cancer referral guidelines, suggesting that RATs should be used flexibly with clinical practice.

Quantitative research is needed to examine the effects of using cancer RATs (such as QCancer) on rates of referral, investigation, diagnosis or overdiagnosis, and whether use of the tool improves patient outcomes compared with current practice.

## Conclusion

This study found a range of barriers and facilitators to implementation of QCancer. While facilitators could encourage the use of the tool, the barriers to implementation should be considered and addressed while implementing the tool in primary care.

## Supplementary material

For supplementary material accompanying this paper visit http://dx.doi.org/10.1017/S1463423621000281. click here to view supplementary material
