# Supplementary material for: Barriers and facilitators to implementing a cancer risk assessment tool (QCancer) in primary care: a qualitative study
Source: Prim Health Care Res Dev. 2021 Oct 7;22:e51. doi: 10.1017/S1463423621000281 (PMC8527274; doi:10.1017/S1463423621000281)
Supplement: Supplementary file 1 [file phcsup.zip › phc.sup/S1463423621000281sup002.docx]

**Supplementary Table S2: Barriers to implementing a cancer risk assessment tool (QCancer) in primary care – details of theoretical framework, codes, key themes and quotations**

| **Consolidated Framework for Implementation Research (CFIR) construct** | **Codes** | | **Key theme** | **Meaning of theme** | **Quotations** | |
| --- | --- | --- | --- | --- | --- | --- |
|  | Service users | Practitioners |  |  | Service users | Practitioners |
| Patient needs & resources | -More time to discuss risk of cancer)  -There may be cost e.g time initially with implementation | -Time and workload pressure | **Additional consultation time** | Perceived additional time required for the extra task of using Cancer risk Assessment Tools (CRATs) could challenge the implementation of CRATs | *“Practitioners in general practice would need more time to use the tool in consultations”* (Service User (SU) 7: individual interview) | *-“It’s more a question of more time really, because at the moment we're in crisis, GPs are in crisis, and the future is very bleak for GPs. Because you come in at 5 in the morning and you get back home in the night and it’s a nightmare really. So we don't want more work. But we can target and do what we need, so unless we really suspect cancer” (P 11[GP]: FG 2)*  *-“the uncertainty and putting all the data manually”* (P 12[GP] : FGD 3)  *-“It should be integrated in our system, systemOne, rather than every time we have to go on Google to get it. Like you put all that information in, it will be difficult, it should be completely automatic, and think of the investigations, people are more likely to use it”* (P 12[GP]: FG 3)  -*“Well I believe if, in the ideal world, you may be aware of that, if there is a push to implement that; I think we should have 15 minutes’ appointments. That will give us a little bit extra time, which is assuming the patient has only one problem, but they have multiple problems in one appointment, I think it will be done well if something like cancer risk assessment, communication, organising of the test that follow, 15 minutes will be ideal*” (P 1[GP]: individual interview) |
| Patient needs & resources | **-**Worry due to investigations  –Anxiety due to referral  -Misinformation of a person’s risk | -Generating unnecessary patient anxiety  --Fear of litigation-issue of negligence (if cancer missed based on QCancer low risk) | **Unnecessary worry relating to cancer investigations** | Perceived unnecessary worry/anxiety related to using CRATs could challenge the implementation of the tools | *-“Some people may not understand and they can be too worried especially if they don't explain that it is just a risk but it is not guaranteed that they will get cancer, then it is not good enough ethically”* (SU 11: individual interview).  -*“I think they may be, have to assess their patients first to see if they were going to promote great anxiety on the patients by using some of these tools. I think emotionally I would be quite distraught and worried, and this is where having another person in there is important I think. But I think until you've got some sort of appointment and then further tests and so forth, you will be in a state of limbo, I think, you know, not being able to concentrate properly”* (SU 6: individual interview) | *-“The thing is if you tell the patient they've got 1% cancer, which is creating unnecessary anxiety”* (P 2[GP]: individual interview)  *-“You can probably make them more worried”* (P 16 [Practice Nurse]: FG 3). |
| Patient needs & resources | -Potential for over referral  -Potential burdening of resources | -Potential for over referral and burden or strain on NHS resources  -Need for sufficient secondary care capacity for rapid assessment | **Over-referral & over burdening of services** | The perceived additional referrals could over-burden services | *-“It could be useful if the right patients are referred but it could also lead to over referral as some people may have a certain risk but will not have cancer after they have been referred and tested*” (SU 17: individual interview) | *-“But on the other side it will put a strain on the NHS; you know what I mean, on the services there. You know, you don't want to over burden the services as well”* (P 4[GP]: individual interview)  -“*We are not just referring but we are using our clinical judgements as well, so we would only refer those patients that need to be referred – so I don’t think there will be over-referrals”* (Practitioner 1 [GP]: FG 1)  -*“I think we should not be worried about resources. Now many more people are dying from cancer so they want more referral and diagnosis earlier. I am not sure using the tool is cheaper but I will say it is good for patient safety because people will be diagnosed earlier if referred earlier and they will be helped earlier*” (P 3[GP]: individual interview) |
| Beliefs & knowledge of individuals involved | - | -Some practitioners may be sceptical | **Practitioner scepticism about using CRATs** | Those sceptical about the new tools may be unwilling to use them | *-* | *-“….until you said this thing, you know initially I was very sceptical about this tool”* (P 3[GP]: individual interview)  -“I believe it will be good to use a cancer risk assessment tool to facilitate earlier diagnosis of cancer, and as you know, earlier diagnosis will help with earlier treatment” (Practitioner 2 [GP]: FG 1)  -“GP experience is more important than tools and guidelines” (P 10[GP]: FG 2)  -*“I think for it to be useful there needs to be some benchmarks for us to really relate with. If you say 7% or 6%, should I worry? I think they need to tell us. So for cancers, unless we have those things it will probably be difficult to use the tool”* (P 12[GP]: FG 3) |
| Complexity | -Everybody should use the same sort of guidelines | -Current risk assessment is based on practitioners’ knowledge of patients' symptoms, cancer risk factors and NICE guideline  -Referral also depends on important single risk factors e.g. age | **Conflict with existing guidelines** | Potential confusion in use with existing tools could challenge the use of CRATs | *-“I think it is good for everybody to have the same sort of guidelines, so to use risk assessment tools everybody should use the same sort of guidelines”* (SU 1: Individual Interview) | *- “I will be quite confused about using the tool. With the NICE guidelines, you couldn't focus on another criterion for any other risk here. I mean there are implications for investigations, referrals…, it has to be very much a repeated approach” (Practitioner 11 [GP]: FG 2)* |
| Complexity | -It doesn't really matter about percentages  - the fact is the symptom is there which is quite worrying | -Some symptoms indicate referral whatever the risk percentage  -High risk prompts further investigation  -Some patients may wish to be investigated at a low 1% risk  -Varying perception of risk from 1-10%, e.g. 4%-10% risk of cancer is high and needs referral with investigations for low risk e.g 1% | **High risk symptoms need referral at any risk** | The tools may not need to be used where symptoms are suggestive of cancer | *-“It doesn't really matter about percentages; I know 1% is less risk. But the fact is the symptom is there, the coughing out of blood, which is quite worrying”* (SU 13: individual interview)  -*“to see whether it is cancer or something else”* (SU 9: individual interview) | -*“..as I said, if I suspect cancer and I put in the tool 1%, 2% doesn't matter to me”* (P 5[GP]: individual interview)  -*“Regardless of what QCancer said I will refer them for investigation with the symptoms. So it doesn't matter 1% or 0%, I will always do one thing, investigation if the symptoms are suggestive of cancer*” (P 11[GP]: FG 2) |
| Beliefs & knowledge of individuals involved | - | QCancer tool not properly understood  Training and education required | **Need for training on how to use the tools** | Training will help practitioners to know more about and understand how to use the tool in patient consultations | - | “*We don’t quite understand how to use that tool. I think we need to have proper education or training on using these tools”* (Practitioner 2 [GP]: FG 1). |
| Reflecting & monitoring | -Need for further investigation of symptoms and risk of cancer  - | -Need to compare with current practice before and after implementing CRATs | **Establishing effectiveness of the tools** | Waiting to pilot and evaluate the tools before rolling them out could delay or challenge full implementation | *-“I think if you are going to roll something out rather than going to everybody I would start with the doctors, see how the doctors do with it after evaluation and then move on to the practice nurses”* (SU 12: individual interview) | *-“We have to make sure that it is better than what we are already doing”* (P 13[GP]: FG 3)  *-“One of the things I think will be really useful is, it's been devised but getting it in real life and then reporting back on that, it will be really useful to know how good a tool it is* (P 1[GP]: individual interview) |

**Supplementary Table S2 (continued)** **Facilitators to implementing a cancer risk assessment tool (QCancer) in primary care – details of theoretical framework, codes, key themes and quotations**

| **Consolidated Framework for Implementation Research (CFIR) construct** | **Codes** | | **Key theme** | **Meaning of theme** | **Quotes** | |
| --- | --- | --- | --- | --- | --- | --- |
|  | Service users | Practitioners |  |  | Service users | Practitioners |
| Relative advantage | -Promotes consistent decision making  - Will promote awareness of cancer symptoms  -Adds to clinician's knowledge and skills  -Useful if combined with clinical knowledge and skills | -Cancer risk assessment tools could aid decision making  -Aid at initial consultation  -For a differential decision or diagnosis  -For cancer screening programmes in general practice  -Those with suspicion of cancer  -For patients with vague or doubtful or borderline symptoms  -Empowering patients in decision making  -Breaking bad news to patients  - Will generate data e.g. for research | **Supporting clinical decision-making** | Participants perceive the benefit of supporting decision making as a facilitator to the implementation of CRATs | *-“a structured way of going through risk”* (SU 7: individual interview)  *- “It will help to make decisions appropriately”* (SU 1: individual interview)  -*“Yes, it is useful because the tool will help the practitioners to ask the right questions, and I know you have just 5 or 10 minutes with the doctor, and if the use of the risk assessment tools helps with the process then I think they should be used”* (SU 18: individual interview) | *-“I think the tool will help to guide the clinician to see the broad level of differential diagnosis. It will also facilitate referral of patients by presenting a quantitative risk value to help explain risk and make a decision” (Practitioner 2 [GP]: Focus Group (FG 1)*  *-“I think somebody where you thought they have a cancer, probably you wouldn't go on a QCancer, and you would do what you do now. I think with people who are at the borderline, I think for these people you might want to use it*” (P 15[GP]: FG 3)  *-“I think one of the ways I can use this tool is when you have got a differential in your mind, how can you put the cancer which may be at the lower end of the spectrum to come on top?”* (P 10[GP]: FG 2)  -“*I will recommend that CRATs like QCancer be available to patients to use before coming to their GP.”* (P 5[GP]: individual interview) |
|  | -Will promote education and awareness of cancer symptoms  -Information about modifying health behaviours  -Identification and modification of cancer risk factors | -Promoting understanding  and behaviour  -Patient education about cancer risk  -Support lifestyle advice and patient motivation to reduce cancer risk  -Encourage patients to check risk and seek advice from GP | **Identifying and modifying health risk behaviours** | The potential benefit of helping to identify and advise people to modify their health risk behaviours is perceived as a facilitator for the implementing CRATs | *“I think it might be just raising awareness, so people realise what's happening, and what can go wrong with them and where the risks are and may be, they can reinforce them where someone else like the young person who has given up smoking it might be used to reinforce by saying well you've got a very low risk so if you've given up smoking carry on with that. Rather than saying you've got a very high risk later on”* (SU 5: individual interview)  -*“to be forewarned is to be forearmed, so they change their life styles such as stopping smoking or drinking alcohol”* (SU 10: individual interview)  -*“It can help to identify the individual's risk, isn't it? Because the input you've given is about your own risk rather than the general population, and if it's done over some years and your risk is increasing they could turn round and say well we need to increase or make more changes in your life style. So if they were to do it every 5 years and if they see that the risk is increasing then maybe they could start giving me some life style advice”* (SU 18: individual interview) | *-“I also feel the tool will help in terms of using the risk generated to advise patients who need behavioural changes. If their risk was small, I would tell them to maintain healthier lifestyles by exercising, eating a healthy diet, less alcohol and to stop smoking if they were smoking. Yes, as I said, this tool can help to empower patients to take control of their risk factors and live healthier lifestyles” (Practitioner 2 [GP]: FG 1)*  *-“They don't understand the risks, you know what I mean? I mean like someone who is a smoker, he is smoking, smoking. You can use this tool to help them modify their life style. People who are refusing referral, you can use the tool to estimate their risk to show and explain to them”* (P 2[GP]: individual interview  -*“I am saying they could also check and then come to us, and they could be helping us as well. If what they regard as risk, you know almost the fact that they've come that might suggest they regard whatever number they've got as something we will be able to discuss with them. We go over it and you could recheck. You could recheck the cancer risk with the patient to see whether you have the same result”* (P 8[GP]: FG 2) |
|  | -Earlier detection of cancer symptoms  -Provides structured risk assessment | -Will help to improved speed of assessment  -Earlier detection and treatment  -More rapid investigation and referral  -Improved outcomes  -Capture of cases missed by two-week wait  -Will generate data e.g. for research | **Improving process and speed of assessment and treatment** | The benefit of helping to improving process of and speed of assessments and treatment is perceived a facilitator to implementing CRATs | *-“it will help with early diagnosis through early detection of cancer risks”* (SU 10: individual interview)  *-“I think my first worry is that I may have cancer and most of us will like to know early so they can get it sorted. But a lot of things can be picked up, can't they, if they spot check risk if you like”* (SU 4: individual interview) | *-“I think when the tool is fully integrated in our IT systems and every practitioner gets familiar with using it, it will be time saving in the long term, as the consultation, the assessments, investigations and referral processes will be faster” (P 1 [GP]: FG 1)*  *-“Well as a tool, it's useful, for helping practitioners' ability to spot cancer or the possibility of cancer at an earlier stage than we could do. You know all emphasis is on cancer care, and GPs are sometimes a bit, a bit stuck to know what to pick as symptoms of cancer”* (P 8[GP]: FG 2)  -*“With everyone on board because we need more investigations, we need more tests quickly”* (P: 11/FG 2) |
|  | -Will help clinicians to know the patient  -Promote individualised patient centred care | -More accurate and specific information for individual patients  -Individualised assessment and care  -Reassurance for both low and high cancer risk  -Reduced complaints from different patients | **Personalising patient care** | The benefit of helping to personalise patient care is perceived a facilitator to CRATs implementation | *“I think it will make the care more patient-centred because you're presenting them with their own risk not a general risk, it's personal to them and it will just make the consultation more patient focused, and I think it will make patients feel more involved in the consultation and just feel more cared for” (SU 12: individual interview).*  -*“So it needs to be about me. I think it’s an excellent idea, the tool. I mean it's dealing with the individual very specifically and there's an opportunity to explain things in more details and delay the inevitable anxiety”* (SU 19: individual interview) | “*Patients* *will go away with a lot more targeted information about their personalised risk of cancer rather than a vague statement”* (P 1[GP]: individual interview) |
