# Supplementary material for: Barriers and facilitators to implementing a cancer risk assessment tool (QCancer) in primary care: a qualitative study
Source: Prim Health Care Res Dev. 2021 Oct 7;22:e51. doi: 10.1017/S1463423621000281 (PMC8527274; doi:10.1017/S1463423621000281)
Supplement: Supplementary file 1 [file phcsup.zip › phc.sup/S1463423621000281sup001.doc]

**Supplementary Table S1: Consolidated criteria for Reporting Qualitative research (COREQ) Checklist**

| **No. Item** | **Guide questions/description** | **Reported on Page** |
| --- | --- | --- |
| **Domain 1: Research team and reﬂexivity** |  |  |
| *Personal Characteristics* |  |  |
| 1. Interviewer/facilitator | Which author/s conducted the interview or focus group? | JNA |
| 2. Credentials | What were the researcher’s credentials? E.g. PhD, MD | MPhil |
| 3. Occupation | What was their occupation at the time of the study? | PhD student |
| 4. Gender | Was the researcher male or female? | Male |
| 5. Experience and training | What experience or training did the researcher have? | JNA had training and experience of using focus groups and individual interviews to collect and analyse qualitative data. |
| *Relationship with participants* |  |  |
| 6. Relationship established | Was a relationship established prior to study commencement? | Yes, the researcher introduced himself and interacted briefly with participants as part of ground preparation for the interviews and focus groups. |
| 7. Participant knowledge of the interviewer | What did the participants know about the researcher? e.g. personal goals, reasons for doing the research | As a PhD researcher with interest in cancer risk assessment tools. |
| 8. Interviewer characteristics | What characteristics were reported about the interviewer/facilitator? e.g. Bias, assumptions, reasons and interests in the research topic | Characteristics of interviewer not reported in the paper, but during the interviews, JNA the interviewer maintained an open mind and ensured his personal views and knowledge did not influence the views of participants. |
| **Domain 2: study design** |  |  |
| *Theoretical framework* |  |  |
| 9. Methodological orientation and Theory | What methodological orientation was stated to underpin the study? e.g. grounded theory, discourse analysis, ethnography, phenomenology, content analysis | Framework Analysis (Richie and Spencer, 1994) as stated on Pages 7. |
| *Participant selection* |  |  |
| 10. Sampling | How were participants selected? e.g. purposive, convenience, consecutive, snowball | By convenience sampling as described on Pages 4. |
| 11. Method of approach | How were participants approached? e.g. face-to-face, telephone, mail, email | Face-to-face individual interviews and focus groups as stated on Pages 4. |
| 12. Sample size | How many participants were in the study? | 36 (19 service users and 17 primary care practitioners) as stated on Page 7. |
| 13. Non-participation | How many people refused to participate or dropped out? Reasons? | There were no dropouts or refusal to participate. |
| *Setting* |  |  |
| 14. Setting of data collection | Where was the data collected? e.g. home, clinic, workplace | In service users’ homes and university interview rooms, depending on service users’ preference. All practitioners were interviewed at their general practices, where they usually work. |
| 15. Presence of non-participants | Was anyone else present besides the participants and researchers? | No |
| 16. Description of sample | What are the important characteristics of the sample? e.g. demographic data, date | As in Table 1: 19 service users (aged 21 to 71 years); 7 males, 12 females; all White British; no one from Black and Minority Ethnic background; 2 had a previous diagnosis of cancer, and the rest had relatives or friends who had a previous diagnosis of cancer.  17 practitioners (aged 33 to 55 years); 13 males, 3 females; 6 White British, 11 of Asian ethnicity. |
| *Data collection* |  |  |
| 17. Interview guide | Were questions, prompts, guides provided by the authors? Was it pilot tested? | Yes |
| 18. Repeat interviews | Were repeat interviews carried out? If yes, how many? | No |
| 19. Audio/visual recording | Did the researcher use audio or visual recording to collect the data? | Yes |
| 20. Field notes | Were ﬁeld notes made during and/or after the interview or focus group? | Yes |
| 21. Duration | What was the duration of the interviews or focus group? | 30 - 45 minutes per interview 45 - 60 for focus group. |
| 22. Data saturation | Was data saturation discussed? | Yes, as stated on Page 7. |
| 23. Transcripts returned | Were transcripts returned to participants for comment and/or correction? | No |
| **Domain 3: analysis and ﬁndings** |  |  |
| *Data analysis* |  |  |
| 24. Number of data coders | How many data coders coded the data? | 2 people (JNA, ANS) coded the data as stated on Page 7. |
| 25. Description of the coding tree | Did authors provide a description of the coding tree? | Yes, page 7. |
| 26. Derivation of themes | Were themes identiﬁed in advance or derived from the data? | Themes were both identified in advance and then from the data using framework analysis as described on Page 7. |
| 27. Software | What software, if applicable, was used to manage the data? | NVivo software as stated on Page 4. |
| 28. Participant checking | Did participants provide feedback on the ﬁndings? | No |
| *Reporting* |  |  |
| 29. Quotations presented | Were participant quotations presented to illustrate the themes/ﬁndings? Was each quotation identiﬁed? e.g. participant number | Yes, in the results on Pages 8-16 and in Supplementary Table S2 |
| 30. Data and ﬁndings consistent | Was there consistency between the data presented and the ﬁndings? | Yes, in the results on Pages 8-16. |
| 31. Clarity of major themes | Were major themes clearly presented in the ﬁndings? | Yes, in the results on Pages 8-16. |
| 32. Clarity of minor themes | Is there a description of diverse cases or discussion of minor themes? | Yes, see discussion of results/themes on Pages 17-23. |

Developed from: Tong A, Sainsbury P, Craig J. Consolidated criteria for reporting qualitative research (COREQ): a 32-item checklist for interviews and focus groups. International Journal for Quality in Health Care. 2007, Volume 19, 349 – 357.
